# Price subsidies increase the use of private sector ACTs: evidence from a systematic review

**DOI:** 10.1093/heapol/czu013

**Published:** 2014-03-14

**Authors:** Alexandra Morris, Abigail Ward, Bruno Moonen, Oliver Sabot, Justin M Cohen

**Affiliations:** ^1^Clinton Health Access Initiative, 383 Dorchester Avenue, Suite 400, Boston, MA, USA and ^2^Slingshot, 67 Riverside Dr. Nairobi Kenya 00100 London, UK

**Keywords:** Subsidy, artemisinin combination therapies, malaria, systematic review

## Abstract

**Background** Although artemisinin combination therapies (ACTs) are the recommended first-line treatment for uncomplicated malaria in most endemic countries, they have been prohibitively expensive in the retail sector where many suspected malaria cases purchase treatment. ACT subsidies seek to stimulate consumer demand for the drugs over cheaper but often ineffective alternatives by reducing their prices. Recent evidence from eight regions implementing such subsidies suggests that they are generally successful in improving availability of the drugs and decreasing their retail prices, but it remains unclear whether these outcomes translate to improved use by patients with suspected malaria.

**Methods** A systematic literature review was conducted to identify reports of experimental or programmatic ACT subsidies to assess the impact of subsidies on consumer use. Relationships between price, use and potential confounding factors were examined using logistic and repeated measures binomial regression models, and approximate magnitudes of associations were assessed with linear regression. In total, 40 studies, 14 peer-reviewed and 26 non-peer-reviewed, were eligible for inclusion in the analysis. The reviewed studies found a substantial increase in private sector ACT use following the introduction of a subsidy. Overall, each $1 decrease in price was linked to a 24 percentage point increase in the fraction of suspected malaria cases purchasing ACTs (*R*^2^ = 0.302). No significant differences were evident in this relationship when comparing the poorest and richest groups, rural vs urban populations or children vs adults.

**Conclusions** These findings suggest that ACT price reductions can increase their use for suspected malaria, even within poorer, more remote populations that may be most at risk of malaria mortality. Whether a subsidy is appropriate will depend upon local context, including treatment-seeking behaviours and malaria prevalence. This review provides an initial foundation for policymakers to make evidence-based decisions regarding ACT price reductions to increase use of potentially life-saving drugs.

**KEY MESSAGES**

- Subsidies increased private sector ACT usage by children under the age of five in all of the studies identified, from 7 percentage points in Nigeria to 49 percentage points in Uganda (both at national scale).
- On average, a 1 dollar reduction in the price of ACTs was associated with a 24 percentage point increase in ACT usage among those of any age, and a 32 percentage point increase among children under five.
- The observed increases in ACT use in the private sector following subsidization appear equitable; subsidies substantially increased ACT use even among the poorest individuals studied, and no significant differences were apparent in ACT use increases between those living in rural vs urban areas.

## Introduction

Increasing the use of artemisinin combination therapies (ACTs), the recommended first-line treatment for uncomplicated malaria in most endemic countries, is a central pillar of the global effort to reduce malaria mortality (WHO 2010). Over the past 8 years, substantial international donor investment has focused on making ACTs widely available in the public sector in many countries in sub-Saharan Africa. However, ACT use is greatly limited by the medicine’s high cost in the private sector, where roughly half of malaria patients in sub-Saharan Africa, and an even higher proportion in Southeast Asia, purchase treatment (WHO 2011). In recent years, ACTs have been found to be more than 10 times more expensive than common but potentially ineffective anti-malarials such as chloroquine and sulphadoxine pyrimethamine (White 2008). As a result, many patients who obtain treatment in the private sector continue to use sub-optimal medicines.

To address this financial barrier and increase access to affordable ACTs, several countries have introduced programmes to subsidize the price of ACTs in the private sector. Most recently, in 2009, the Global Fund to Fight AIDS, Tuberculosis and Malaria (the Global Fund) launched the Affordable Medicines Facility—malaria (AMFm), an international financing mechanism piloted in eight territories—such as Nigeria, Niger, Madagascar, Uganda, Ghana, Kenya, Tanzania and Zanzibar. The initial phase of the AMFm was comprised of three elements: (1) price reductions through negotiations with ACT manufacturers, (2) a buyer subsidy offered through a co-payment at the manufacturer level of the supply chain and (3) supporting interventions to promote appropriate use of ACTs. The AMFm provided a roughly 90% co-payment for ACTs ordered by both public and private sector buyers in an effort to reduce the price to consumers.

A policy review published in 2011 suggested ACT subsidies were successful in improving availability of the drugs and decreasing their retail prices (Schäferhoff and Yamey 2011). However, little data were available on whether these intermediary outcomes translated to an improvement in the fraction of suspected or confirmed malaria patients who actually received an ACT. Following the launch of the AMFm, an independent evaluation was commissioned to assess changes in ACT price, availability, market share and use (Arnold *et al.* 2012). An extensive analysis was conducted to measure the changes in these outcomes at national scale across the pilot countries before and after the AMFm intervention. Due to cost and complexity, however, the Global Fund decided to not conduct measurement of ACT use as part of that evaluation. Instead, the evaluation considered existing household surveys available through Demographic Health Surveys (DHS) and other sources, including surveys conducted by ACTwatch Group of Population Services International (ICF Macro and LSHTM 2012). The report on ACT use concluded that three of the five countries with available evidence demonstrated at least a five percentage point increase in ACT use pre- and post-subsidy introduction.

This investigation seeks to provide additional evidence on how ACT use changes when prices are subsidized, drawing on additional data from operational research measuring the impact of the AMFm, as well as from prior experimental and programmatic subsidies. A comprehensive review of ACT use data in all areas where an ACT subsidy has been introduced and evaluated was conducted, and these data were used to assess the empirical relationship between ACT use and the proximal factors of ACT affordability and availability. This additional evidence can inform policymakers and funders on if and how interventions to reduce the price of ACTs can enable malaria endemic countries to reach the Roll Back Malaria Partnership goals of achieving universal access in the private sector (RBM 2011).

## Methods

### Literature review

A systematic literature review was conducted to identify studies that examined subsidies for artemisinin-based drugs in the private sector according to PRISMA reporting protocol guidelines (Liberati *et al.* 2009). The private sector was defined as private healthcare facilities, pharmacies and over-the-counter or retail shops. PubMed/Medline and EMBASE were searched in May 2013 for the key words ‘subsidy’ (or ‘subsidi*’) and a MESH heading for ‘malaria’. Non-peer-reviewed evaluations were identified through targeted outreach to investigators of relevant articles and key institutions. Studies were included if they met the following eligibility criteria: (1) the study assessed a financial subsidy in the private sector for an ACT between 1990 and May 2013, (2) study participants were anti-malarial drug customers or users and (3) the study measured whether or not a suspected malaria case received or an anti-malarial customer purchased an ACT. Studies were excluded if no ACT subsidy intervention was introduced, the subsidy was implemented only in the public sector or if there was no attempt to measure ACT use as part of the assessment.

All ACT subsidies described in the reviewed literature were categorized as either corresponding to (1) the AMFm, (2) an experimental subsidy or (3) a non-AMFm, non-experimental programmatic subsidy, typically implemented by the government or an non-governmental organizations. These categories were selected to examine the effect of the AMFm specifically, to observe rigorously measured impact of subsidization in research studies that compared changes against controls and to review evidence on the impact of prior programmatic subsidies. Any information provided on anti-malarial use or purchasing behaviour, patient characteristics and ACT stocking and pricing in retail outlets or health facilities was recorded. The ACT stocking and pricing data were used to look for associations between these measures and use rates.

ACT use has been defined as both (1) the proportion of patients with fevers (or suspected malaria illness) who receive an ACT (Kangwana *et al.* 2011) and (2) the proportion of anti-malarial recipients who receive an ACT (Talisuna *et al.* 2012). The second definition is more appropriate for contexts where anti-malarial use rates may be changing as a result of factors unrelated to the drug subsidy and may also be more appropriate for evaluating the impact of subsidies given that their stated goal is typically to replace ineffective alternative treatment with ACTs rather than to increase the use of anti-malarials in general (Institute of Medicine 2004). If, however, a subsidy does cause changes in the overall envelope of anti-malarial use, this change would not be captured by the second metric. The second narrower definition is primarily used here, although the first is also presented when it is the only metric provided by the reviewed sources.

Two principal methods to measure the fraction of anti-malarial users who take ACTs are used in the literature. The first involves surveys of the community at the household level. This approach is useful because it allows understanding of ACT use from any source, whether public or private, and it allows population-representative measures to be estimated. However, these household surveys are subject to recall biases (Boerma *et al.* 1991), and as drug choice is not directly observed, ACT use rates may not be represented accurately. The second approach involves interviewing customers as they leave drug-dispensing outlets. These ‘exit interviews’ are typically conducted only on a few days outside a select set of outlets, and may or may not be truly representative of the entire region. Since each of these approaches has advantages and limitations in providing a robust measurement of the target indicator, both are reviewed here; clear differentiation is made between the two throughout this review so that the results can be evaluated accordingly.

### Data analysis

Evidence for the impact of subsidies on use was reviewed for each of the three categories of subsidy. Most studies reported the results of cross-sectional surveys conducted at time points before, during and/or after subsidy implementation, permitting examination of how ACT use changed over time. Experimental subsidies additionally provided measurements of use in control groups, allowing attribution of impact to the subsidy specifically. These trends were examined both overall and, where possible, specifically in the private sector. In the public sector, ACT scale up has occurred in many countries through implementation of Global Fund grants and other interventions (WHO 2011). However, no known private sector interventions at national scale were implemented during this period other than the subsidies under consideration.

Where possible, trends were examined within specific subgroups of interest, including children under five, those of lowest socioeconomic status (SES) and those living in rural areas. SES was measured by comparing the highest and lowest wealth index quintiles, generated from principal components analysis of assets by ACTwatch using demographic and health survey methodology. Rurality was considered as a dichotomous rural/urban variable defined separately by each study. Differences in use and increases in use during subsidies between high- and low-SES groups and those in urban and rural locations were compared statistically using logistic regression models.

In a second set of analyses, all studies reporting not only use but also stocking of ACTs and sales prices were compiled to specifically examine the linkage between commonly measured indicators at the retail level and the important but less commonly evaluated impact on use. The relationship between the proportion of outlets stocking ACTs and the average price of ACTs at those outlets was explored using repeated measures logistic regression models with the GENMOD procedure in SAS v9.2. The outcome of this model was whether or not an outlet stocked ACTs. Separate models were run for ACTs for children under five and ACTs for any age. The predictor variable for these models was the average price of ACTs for children under five and for any age, respectively. An exchangeable covariance structure was specified in each model to account for within-survey correlation. Because the resulting odds ratios (ORs) from these models are difficult to interpret (Davies *et al.* 1998), ordinary least squares regression models were also fit to the same data, regressing the fraction of outlets stocking ACTs on the mean ACT price. The resulting linear fit provided a readily interpretable estimate of the average magnitude of the relationship between the two variables. The same approach was then used to examine the relationships between the stocking of ACTs, ACT price and the proportion of anti-malarial customers choosing to purchase them.

## Results

**Figure 1:**
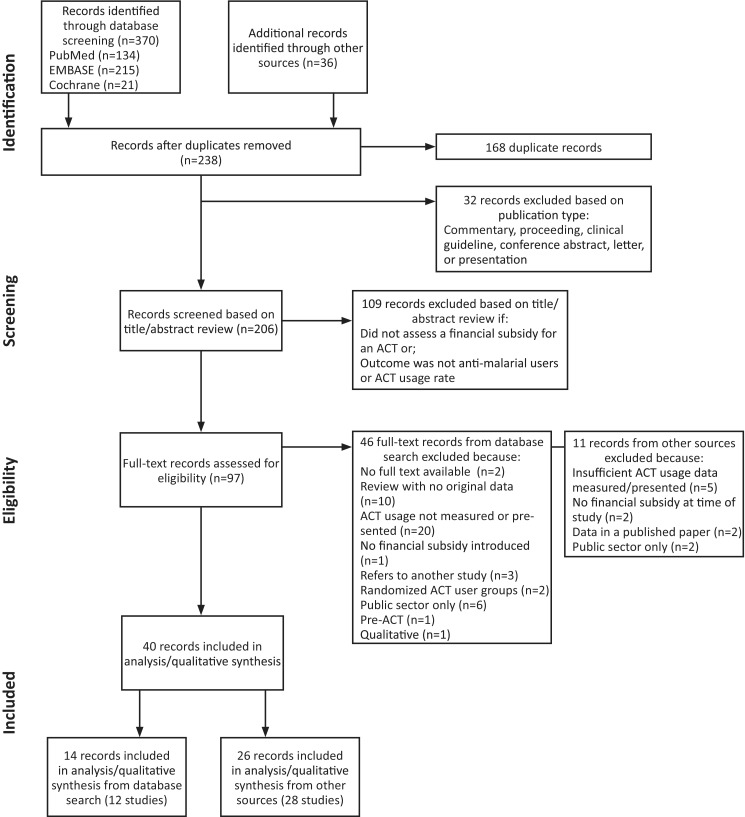
Literature flowchart of ACT subsidies measuring use.

The literature search yielded 370 published, peer-reviewed studies. A total of 206 appeared relevant and were screened by abstract review, of which 97 studies were then selected for full-text review. In addition to the studies identified through the database search, 36 studies were included that were identified through non-peer-reviewed literature or targeted outreach to investigators. In total, 40 studies were eligible for inclusion in the analysis and qualitative synthesis. Of these, 14 were peer-reviewed publications, and 26 were non-peer-reviewed literature (Figure 1). These reports described 10 experimental subsidies in eight countries, non-AMFm programmatic subsidies in nine countries and AMFm subsidies in eight pilots. However, only four experimental subsidies, three programmatic subsidies and five of the eight AMFm pilot subsidies measured ACT use and were therefore included in the quantitative analysis (Supplementary Table S1).

### Experimental subsidies

**Figure 2:**
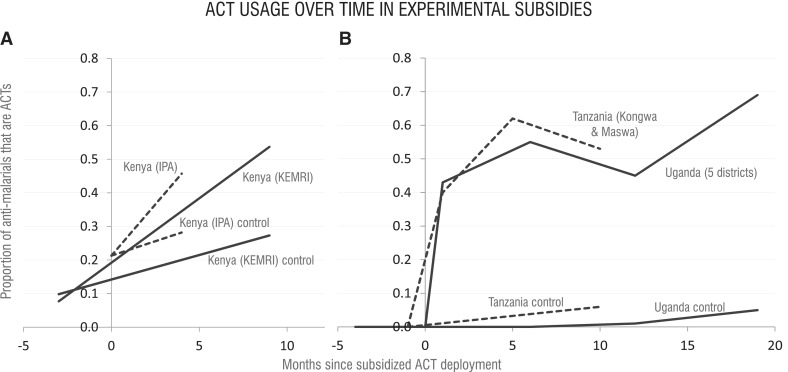
Summary of experimental studies with controlled use data. The fraction of suspected malaria patients receiving ACTs over time in the subsidized and non-subsidized study groups are shown according to **(A)** household surveys of all febrile household members in the rural districts of Busia, Mumias and Samia Kenya (dashed), and febrile children under five in rural Busia, Butere-Mumias and Teso Districts, Kenya (solid). **(B)** Exit interviews with anti-malarial purchasers for children under five in three districts of Tanzania (dashed) and five districts of Uganda (solid). Studies in **(A)** represent the combined private and public sectors **(B)** received them in private outlets only.

Four studies included data on ACT use after subsidization in experimental groups compared with unsubsidized controls (Figure 2). Two of these experiments compared household survey responses regarding the fraction of febrile episodes treated with ACTs (Figure 2A) (Kangwana *et al.* 2011; Cohen *et al.* 2012a,b), while the other two compared the fraction of anti-malarial purchasers receiving ACTs vs other anti-malarials (Figure 2B) (Sabot *et al.* 2009a,b; Cohen *et al.* 2010; Talisuna *et al.* 2012). All four studies found the fractions reporting receiving or purchasing ACTs were significantly higher when subsidies were implemented.

### Non-AMFm programmatic subsidies

Nine programmatic subsidies implemented prior to the AMFm were identified; however, only three subsidies included data on ACT use. In Cambodia, access to subsidized ACTs was scaled up at private retail outlets in 2002. Before the national scale up, household survey results indicated that only 8% of anti-malarial recipients received ACTs (Yeung *et al.* 2011); surveys unrelated specifically to the subsidy found 13% receiving ACTs in 2006 (PSI 2006), 65% in 2009 (ACTwatch Group, Population Services International 2009) and 68% in 2011 (ACTwatch Group and Population Services International 2011). In 2007 and 2008, the Tanzanian government expanded access to subsidized ACTs through private but accredited drug dispensing outlets. Under the subsidy, use of ACTs in the private sector increased from 0% of anti-malarial drugs purchased for children under five to 10% almost a year after subsidy implementation (Simba and Kakoko 2012). In Kenya, a subsidy was launched in 2007 to make ACTs available to Community and Family Wellness shops in three rural districts. In 2006, household surveys found that 15% of patients receiving anti-malarials in either the private shops or public health facilities received ACTs. After 15 months of the intervention, this fraction increased to 42%, with 9% of those receiving ACTs obtaining them from the subsidized shops (Sabot *et al.* 2009a,b).

### The AMFm

**Figure 3:**
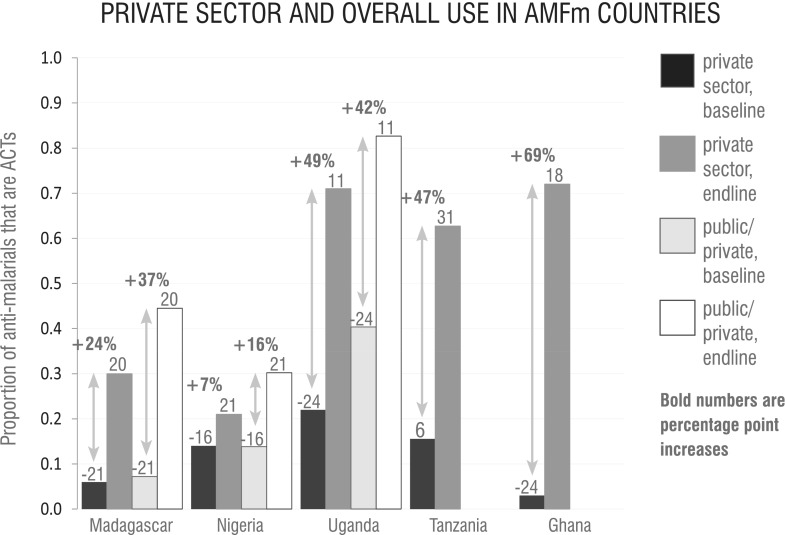
Of those obtaining anti-malarials from the private sector, the proportion that obtained an ACT by country before and during implementation of the AMFm. Data are from ACTwatch surveys in Madagascar, Nigeria, and Uganda and contemporary surveys in Tanzania and Ghana. The timing of each survey with respect to the beginning of the ACT subsidy is given in months in the numbers above each bar. All results are from household surveys except in Tanzania and Ghana, where figures represent the results of exit interviews with anti-malarial purchasers.

The ACTwatch Group collected comparable data on ACT use in Madagascar, Nigeria and Uganda, with surveys conducted 1–2 years before implementation of the subsidy and again after almost 2 years of subsidization in Nigeria and Madagascar and ∼1 year in Uganda (Figure 3) (ACTwatch Group and ABMS/Benin *et al.* 2012). In addition, reports were identified reporting ACT use in Ghana, Tanzania, Uganda and Madagascar following implementation of the AMFm (Quakyi *et al.* 2012; P. Yadav *et al.*, unpublished data). All reports described increases in ACT use after implementation of the subsidy. At the time of analysis, no reports were available on the subsidy’s effect in Zanzibar, Kenya or Niger.

**Figure 4:**
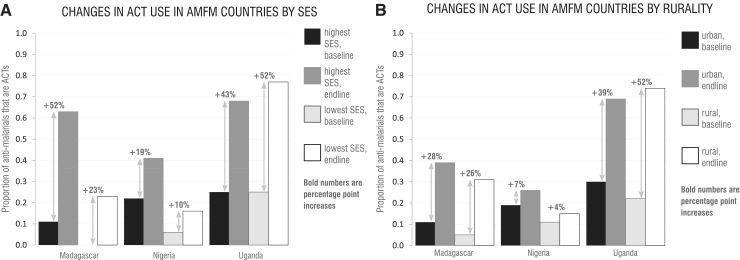
Changes in ACT use in AMFm countries by rurality and SES. (A) Increases in use of ACTs in the private sector by children under five in the lowest and highest SES groups reported in ACTwatch household surveys before and after implementation of the AMFm in Madagascar, Nigeria and Uganda. (B) Increases in use of ACTs by children under five in urban and rural areas as reported in ACTwatch household surveys before and after implementation of the AMFm in Madagascar, Nigeria and Uganda.

The increases in use observed by ACTwatch household surveys of children under five in Madagascar, Nigeria and Uganda varied within SES groups and within urban and rural strata between baseline and endline survey rounds (Figure 4). These classifications reflect categorizations made by the ACTwatch Group in their reports on each country (ACTwatch Group and ABMS/Benin *et al.* 2012).

### Relationships between ACT use, stocking and price

**Figure 5:**
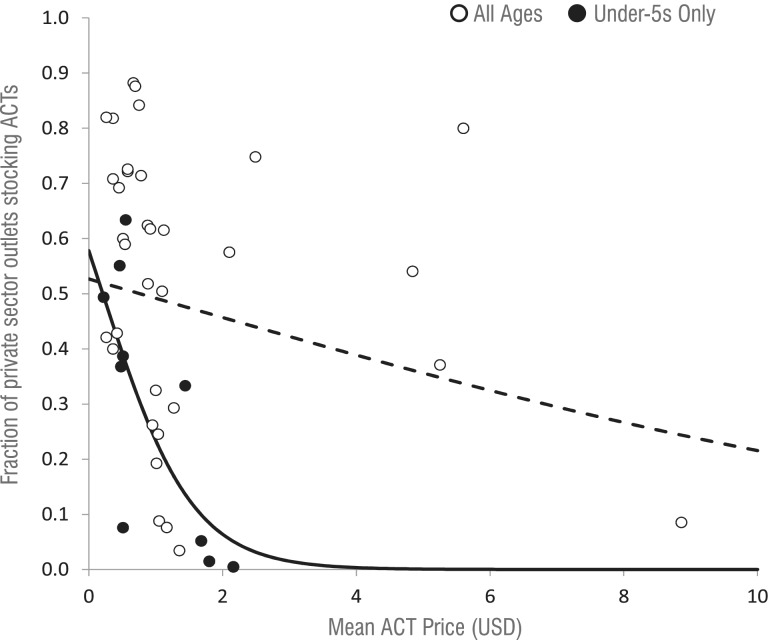
ACT prices and stocking in the private sector. The dashed curve line is the logistic trend for all ages, and the solid line is the logistic trend for under-fives only. Both lines represent the trend without the outlier point, a region in Madagascar with high-ACT stocking and prices.

Figure 5 depicts all reviewed reports that presented data on both the fraction of private sector outlets found to be stocking ACTs and the price of the ACTs in those locations at proximal time periods. Accounting for correlation between outlets measured in the same survey, the price of ACTs for any age group was negatively but not significantly associated with ACT stocking (OR = 0.870, *Z* = −1.66, *P* = 0.098). However, each $1 increase in the price of a paediatric ACT was associated with an OR for stocking of 0.223 (*Z* = −2.62, *P* = 0.009). Ignoring correlation, linear regression output suggests that this $1 increase in price is associated with a 3.49% decrease in the fraction of outlets stocking any ACT (*R*^2^ = 0.051), and a 23.5% decrease in the fraction of outlets stocking a paediatric ACT specifically (*R*^2^ = 0.532).

**Figure 6:**
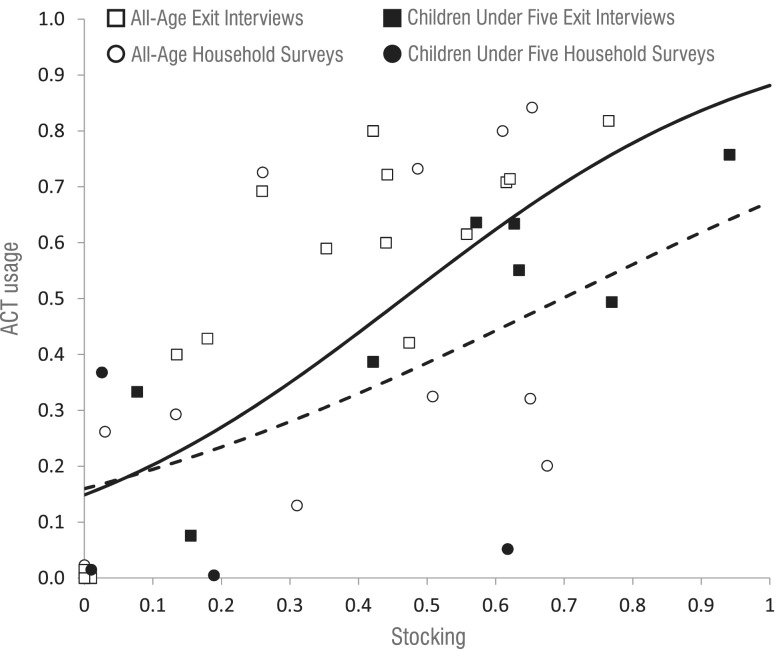
ACT stocking and use in the private sector. Relationship between stocking of ACTs in private sector or outlets and the fraction of anti-malarial users that receive ACTs, by all ages and by children under five only. The dashed line is the trend for all ages, and the solid line is the trend for under-fives only.

In Figure 6, all reviewed studies are depicted that recorded data on both ACT stocking in the private sector and their use in the same regions at proximal time periods. A repeated measures regression model suggests that, on average, each 10% increase in stocking is associated with an OR for ACT use of 1.27 (*Z* = 3.82, *P* < 0.001) among all ages. For children, the OR is 1.46 (*Z* = 3.63, *P* < 0.001). Ignoring correlation between measurements from the same surveys, this 10% increase in stocking is associated with a 5% increase in the fraction using ACTs (*R*^2^ = 0.363) among all ages, and an 8% increase in the fraction of children using ACTs (*R*^2^ = 0.462).

**Figure 7:**
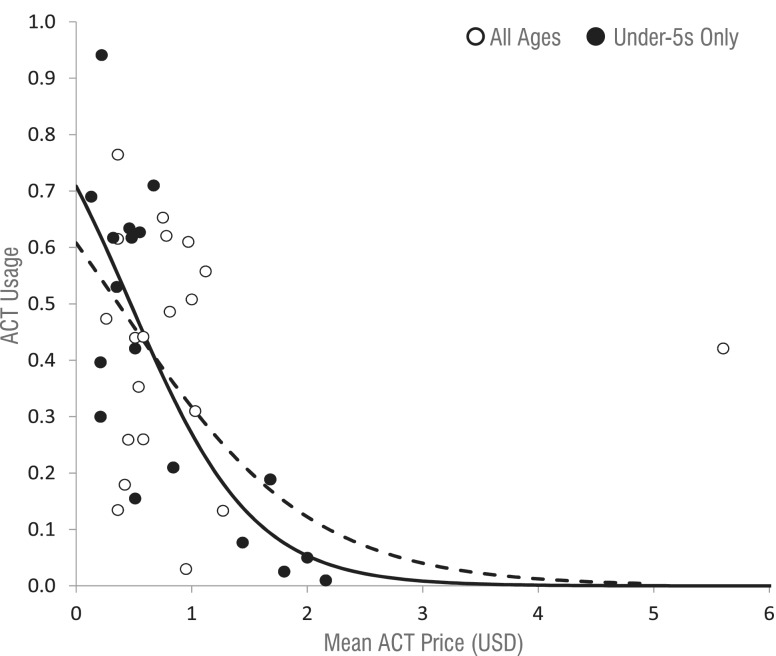
ACT prices and use in the private sector. The dashed line is the logistic curve for all ages, and the solid line is the logistic trend for children under 5 years of age. Both trend lines do not include the high-priced region of Madagascar.

Finally, a negative association was found between mean ACT price and use in the same regions (Figure 7). The overall trend was strongly influenced by a single outlier from Madagascar, where price was >5 dollars. Excluding this point from analysis and accounting for correlation, on average a $1 increase in ACT price was associated with an OR for use of 0.30 (*Z* = −3.02, *P* < 0.001) among all ages, and 0.153 (*Z* = −6.29, *P* < 0.001) among children. Ignoring correlation and again excluding the outlier, a $1 decrease in price was associated with a 24 percentage point increase in ACT use among those of any age (*R*^2^ = 0.302). Restricting only to surveys measuring price for paediatric drugs and use by children under five, a $1 decrease in ACT price was associated with a 32 percentage point increase in ACT use (*R*^2^ = 0.594).

## Discussion

The results of this review suggest that reducing the retail price of ACTs can successfully increase ACT use, although evidence from rigorous studies remains scant. Available results from experimental studies, programmatic subsidies and the AMFm all consistently demonstrate a negative association between ACT use and price. In most non-experimental examples, the lack of controls for comparison means it is impossible to assess the attributable impact precisely, but in the absence of other major interventions aimed at increasing use among private sector treatment-seekers, it is likely that subsidy implementation plays an important role.

The influence of subsidies on ACT use appears consistent within subgroups such as children under five and more remote and poorer populations. Contrary to some stated concerns (Oxfam 2012), this finding suggests that ACT price reductions may have substantial impact on populations that have been identified as being most at risk of malaria mortality (Barat *et al.* 2004). With as many as 60% of young children in areas with the highest burden of malaria obtaining treatment through private channels (Cohen *et al.* 2012a,b), these findings suggest that subsidies may be a useful tool for increasing overall ACT coverage among vulnerable populations not currently reached by the public health system.

This review empirically demonstrates significant relationships between lower ACT prices and their increased availability at retail outlets, between increased ACT availability and a higher fraction of consumers who receive them, and, transitively, between lower ACT prices and their increased use. It is not possible to derive causality from these associations, and it is possible that increased stocking of ACTs is driven by greater consumer demand, or that price reductions are driven by increased availability of the drugs at competing outlets. Nevertheless, these associations, in combination with the available evidence from controlled experimental studies and historical experience, should suggest to policymakers that subsidizing ACTs is likely to result in a higher fraction of treatment seekers receiving them.

This analysis did not examine whether increases in ACT use were specifically among individuals with true malaria infection, although understanding this relationship will be critical for evaluating the true public health significance of increased ACT use (Cohen *et al.* 2012a,b). One of the primary concerns with implementing a subsidy is that low-cost ACTs may be given to patients whose symptoms are not truly caused by malaria infection (Moon *et al.* 2009; Kamal-Yanni *et al.* 2012). To mitigate this risk, ACT subsidies might be considered along with other interventions such as distribution of subsidized diagnostic tests, as has been conducted in Cambodia (Yeung *et al.* 2011). Alternatively, ACT subsidies may be reserved for places where nearly all treatment seekers have a high probability of being infected with malaria (Cohen *et al.* 2012a,b).

It will be important for policymakers to weigh the costs and benefits of implementing a subsidy against those of other potential interventions, particularly in light of constrained resources for malaria programmes (WHO 2013). The results of this investigation provide an initial basis for evaluating the impact of subsidizing ACTs on the fraction of suspected malaria cases who receive them. Results, though based on a limited evidence base, suggest that a 1 dollar reduction in ACT price may increase the fraction of suspected malaria cases seeking treatment in the private sector who receive ACTs instead of other drugs by ∼24 percentage points. Evaluating whether such an investment is worthwhile requires weighing context-specific factors including the fraction of suspected malaria cases who treat outside the formal health sector and the prevalence of malaria infection in this population (Cohen *et al.* 2012a,b).

This review has important limitations. Each of the types of studies reviewed has specific weaknesses: the experimental studies were in limited geographies and controlled environments that may not be fully representative of programmatic conditions, while the programmatic findings tended not to have controls nor proximal baselines, making it impossible to understand the counterfactual scenario of how ACT usage might have changed in the absence of a subsidy. Several of the AMFm evaluations included in the analysis were conducted shortly after the implementation of the subsidy, making it difficult to ascertain the total magnitude of the effect of the intervention. In some cases, such as Uganda, the implementation of the subsidy was significantly delayed, and the evaluation period covered just 11 months. Finally, treatment outcomes are the product of a complex series of factors including social norms, supply chains and interactions between sellers and customers, and it is likely that results of a given subsidy intervention will vary significantly from place to place. Despite these limitations, the preponderance of evidence reviewed here is suggestive that subsidies are likely an effective tool available to policymakers for increasing ACT use outside the formal public health sector.

The Roll Back Malaria Partnership has set a global target of ensuring 100% of confirmed cases receive effective malaria treatment through the private sector by 2015. This review of the available literature suggests ACT subsidies may be an effective tool for equitable progress towards that goal. These results provide an initial foundation for local and global policymakers to make evidence-based decisions regarding whether to implement future ACT price subsidies for reduction of malaria mortality in appropriate contexts.

## Supplementary Data

Supplementary data are available at *HEAPOL* online.

## Funding

This work was supported by the Bill and Melinda Gates Foundation.

## Supplementary Material

Supplementary Data

Translated Abstracts
