# Supplementary material for: Price subsidies increase the use of private sector ACTs: evidence from a systematic review
Source: Health Policy Plan. 2014 Mar 14;30(3):397–405. doi: 10.1093/heapol/czu013 (PMC4353896; doi:10.1093/heapol/czu013)
Supplement: Supplementary Data [file supp_30_3_397__index.html]

Price subsidies increase the use of private sector ACTs: evidence from a systematic review — Price subsidies increase the use of private sector ACTs: evidence from a systematic review — Supplementary Data 

# Price subsidies increase the use of private sector ACTs: evidence from a systematic review

## Supplementary Data

files

**Files in this Data Supplement:**

- Supplementary Data - pdf file
- Supplementary Data - pdf file
- Supplementary Data - pdf file
- Supplementary Data - pdf file
- Supplementary Data - pdf file
- Supplementary Data - pdf file
- Supplementary Data - pdf file
- Supplementary Data - xlsx file
