# Supplementary figures and images for: Price subsidies increase the use of private sector ACTs: evidence from a systematic review
Source: Health Policy Plan. 2014 Mar 14;30(3):397–405. doi: 10.1093/heapol/czu013 (PMC4353896; doi:10.1093/heapol/czu013)

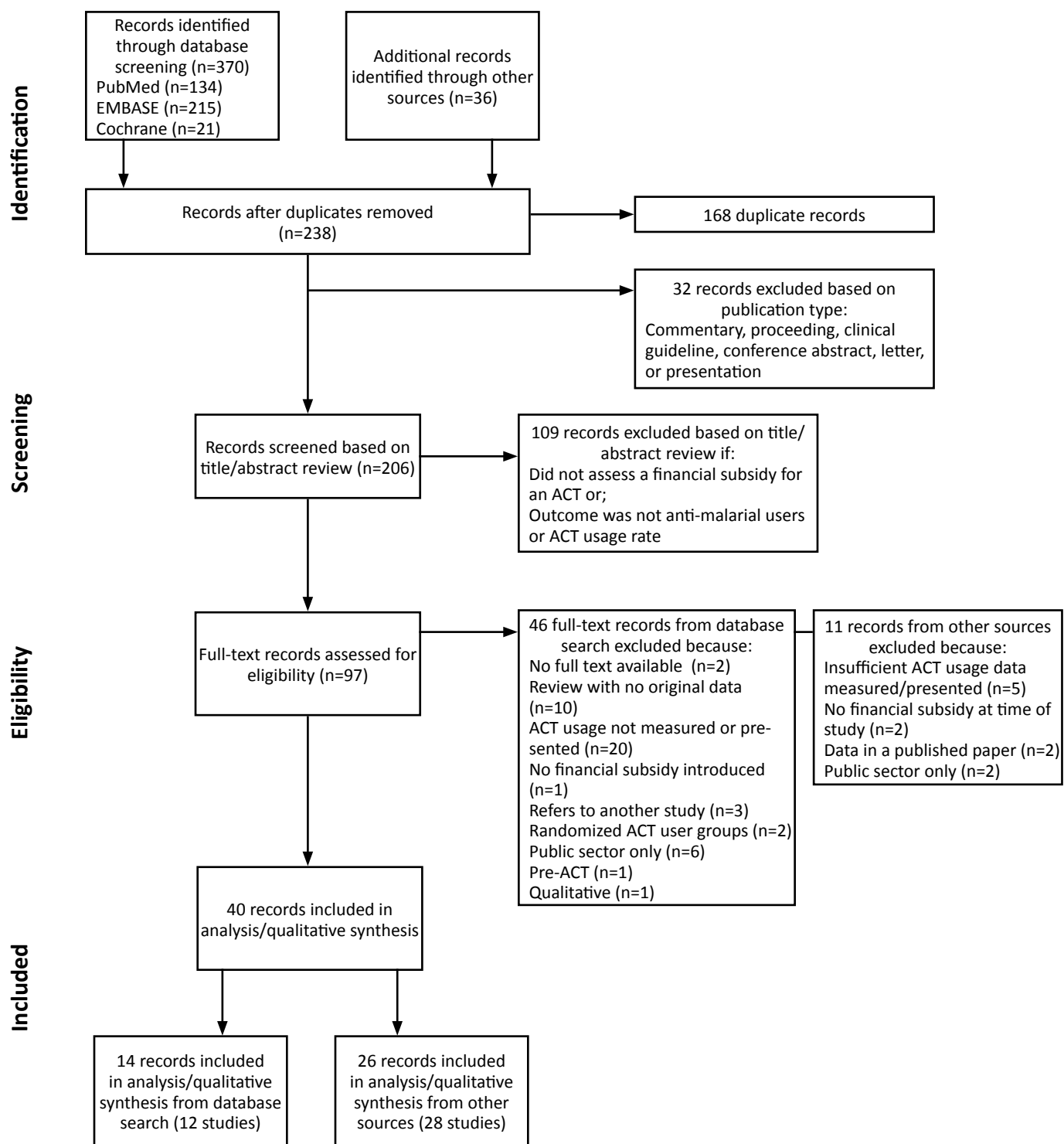

Supplement: Supplementary Data [file supp_czu013_Figure1.pdf]

## ACT USAGE OVER TIME IN EXPERIMENTAL SUBSIDIES

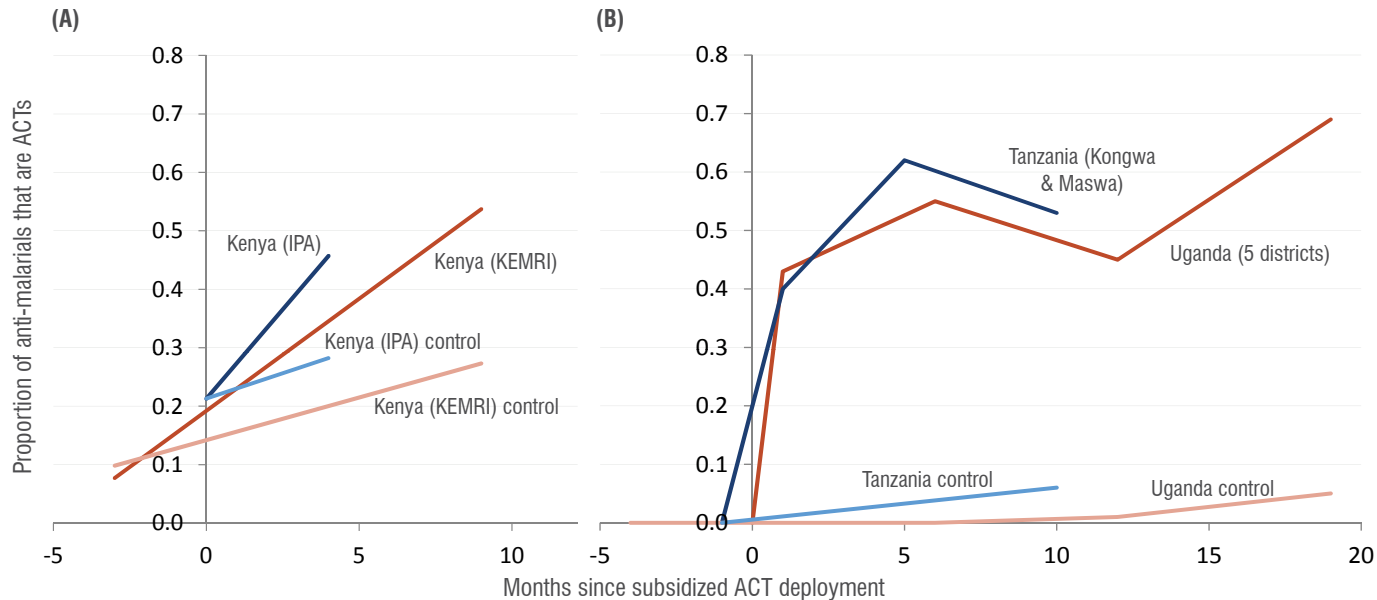

Supplement: Supplementary Data [file supp_czu013_Figure2.pdf]

## PRIVATE SECTOR AND OVERALL USE IN AMFm COUNTRIES

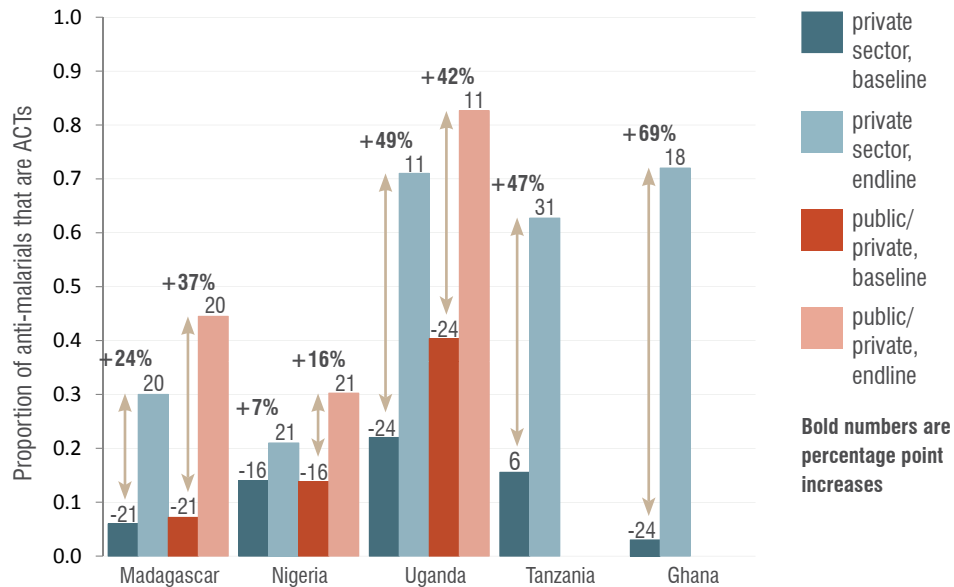

Supplement: Supplementary Data [file supp_czu013_Figure3.pdf]

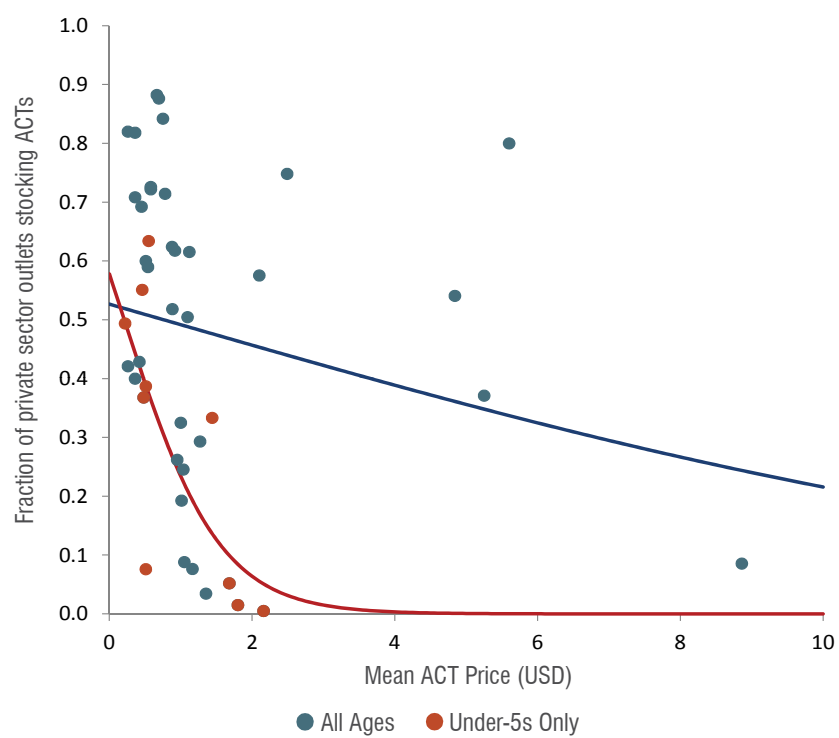

Supplement: Supplementary Data [file supp_czu013_Figure5.pdf]

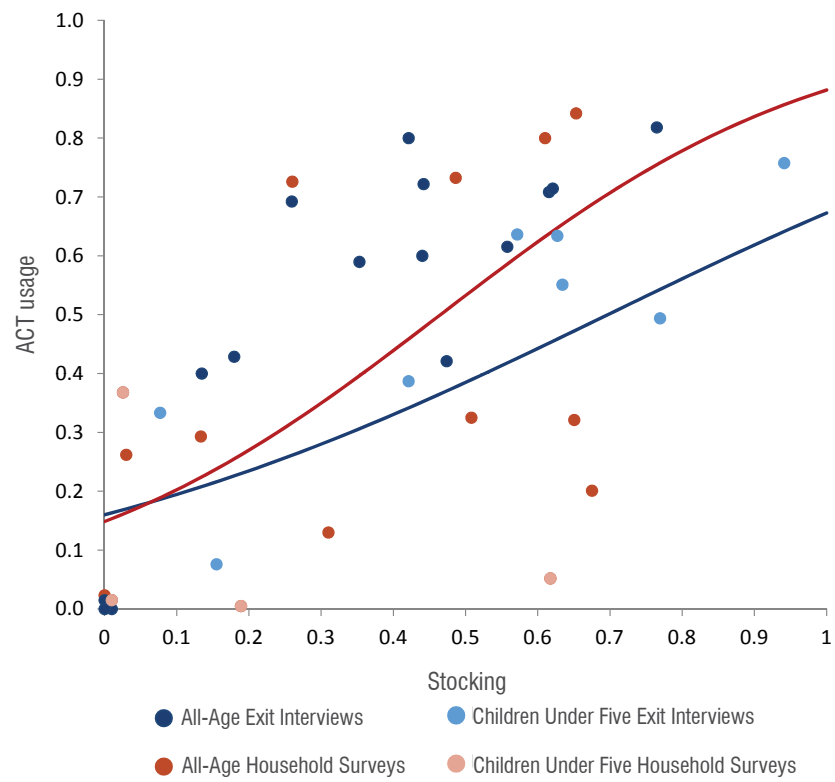

Supplement: Supplementary Data [file supp_czu013_Figure6.pdf]

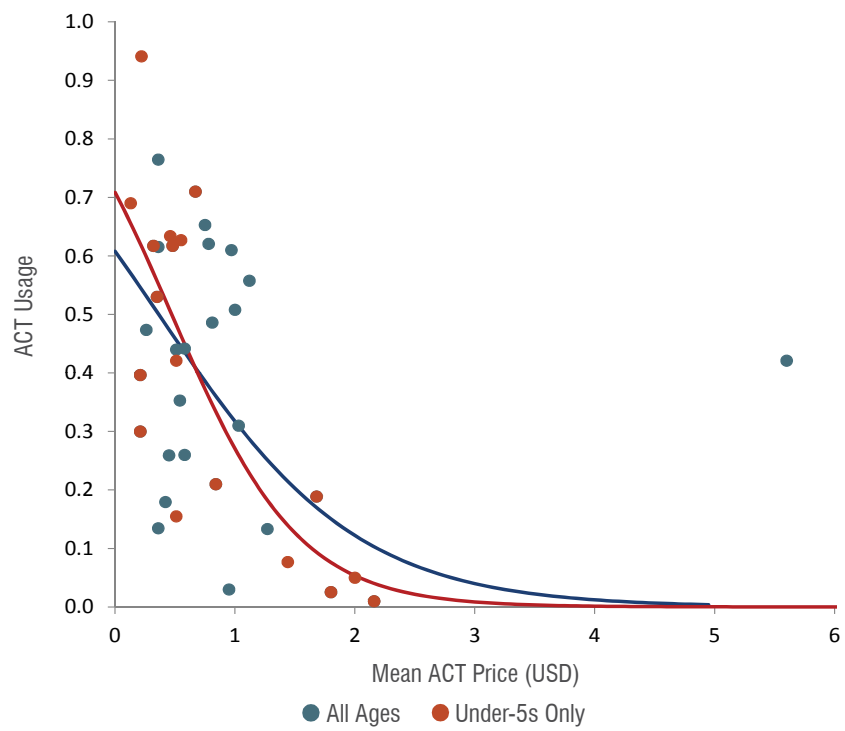

Supplement: Supplementary Data [file supp_czu013_Figure7.pdf]
