# Supplementary material for: Price subsidies increase the use of private sector ACTs: evidence from a systematic review
Source: Health Policy Plan. 2014 Mar 14;30(3):397–405. doi: 10.1093/heapol/czu013 (PMC4353896; doi:10.1093/heapol/czu013)
Supplement: Supplementary Data [file supp_czu013_Figure4.pdf]

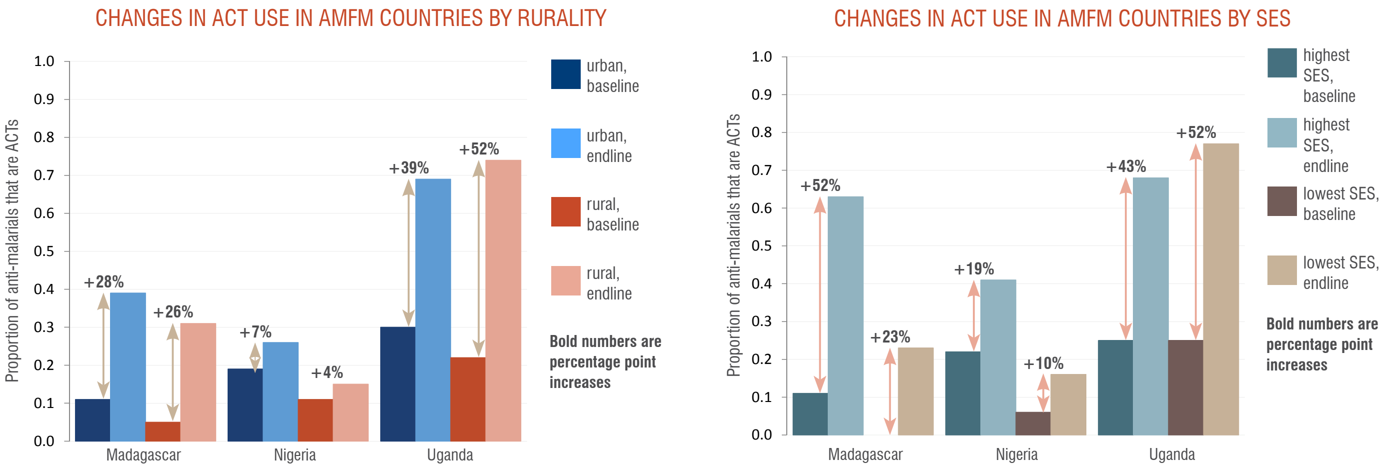

Figure 4. (A) Increases in use of ACTs in the private sector by children under five in the lowest and highest SES groups reported in ACTwatch household surveys before and after implementation of the AMFm in Madagascar, Nigeria, and Uganda. (B) Increases in use of ACTs by children under five in urban and rural areas as reported in ACTwatch household surveys before and after implementation of the AMFm in Madagascar, Nigeria, and Uganda.
