# Supplementary material for: Price subsidies increase the use of private sector ACTs: evidence from a systematic review
Source: Health Policy Plan. 2014 Mar 14;30(3):397–405. doi: 10.1093/heapol/czu013 (PMC4353896; doi:10.1093/heapol/czu013)
Supplement: Translated Abstracts [file supp_30_3_397_v2_index.html]

Price subsidies increase the use of private sector ACTs: evidence from a systematic review — Translated Abstracts 

# Price subsidies increase the use of private sector ACTs: evidence from a systematic review

## Translated Abstracts

files

**Files in this Data Supplement:**

- Chinese Abstract - pdf file
- French Abstract - pdf file
- Spanish Abstract - pdf file
