# Supplementary material for: Price subsidies increase the use of private sector ACTs: evidence from a systematic review
Source: Health Policy Plan. 2014 Mar 14;30(3):397–405. doi: 10.1093/heapol/czu013 (PMC4353896; doi:10.1093/heapol/czu013)
Supplement: Translated Abstracts [file supp_czu013_czu013_Chinese.pdf]

价格补贴增加了私立机构青蒿素联合疗法的使用：系统性回顾的实证研究

研究背景：尽管青蒿素联合疗法在大多数流行疟疾的国家都被当作治疗无并发症疟疾的首选疗法，在零售行业中它昂贵的价格却使很多疑似患者无法购买。青蒿素补贴的目的在于通过降低价格来刺激消费者的消费，以取代其他一些虽然较为便宜但是效果不好的替代药物。从八个地区近期的证据来看，这种补贴总得来说提高了青蒿素的普及和促进了价格的降低，但是补贴是否增加了疑似患者的使用还不明确。

研究方法和结果：我们通过对关于青蒿素补贴的试验或项目报告开展系统的文献研究来评估消费者的使用效果。通过重复测量的双变量逻辑回归模型来研究价格、使用以及可能混淆的因素之间的关系，并用线性回归估计了相关系数的大概级数。最后总共有 40 个研究被用在我们的分析中，其中包括了 14 个同业评议和 26 个非同行评议的研究。这些研究表明，在实施了补助之后，青蒿素在私人机构中的使用得到了显著增加。总的来说，价格每下降一美元，疑似疟疾患者这部分人中购买青蒿素的人数就提升 24 个百分点（R square=0.302）。在贫穷与富裕，乡村与城镇以及儿童与成人之间的差别并不显著。

研究结论：这些研究结果表明青蒿素价格的降低可以增加疑似疟疾患者对其的使用，甚至是在更易因为疟疾而致死的较为贫穷和偏远的人群中。补助的合适与否取决于当地的环境，包括人们的就医行为和疟疾的流行性。本文为决策者们做出降低青蒿素这种可以拯救人性命的药物价格来增加其使用的决定提供了实证基础。
