# Supplementary material for: Price subsidies increase the use of private sector ACTs: evidence from a systematic review
Source: Health Policy Plan. 2014 Mar 14;30(3):397–405. doi: 10.1093/heapol/czu013 (PMC4353896; doi:10.1093/heapol/czu013)
Supplement: Translated Abstracts [file supp_czu013_czu013_French.pdf]

Les prix subventionnés augmentent l’usage d’ACT du secteur privé: conclusion provenant d’une revue systématique

Accepté le 4 février 2014

Même si, dans la plus part des pays endémiques, les thérapies combinées à base d'artémisinine (ACT) sont les premiers traitements recommandés pour les cas de paludisme sans complication, ils sont extrêmement chers à acquérir au détail dans les zones où beaucoup de cas suspects doivent être traités. Les ACT subventionnées cherchent à stimuler, par une baisse des prix, la demande de médicaments alternatifs moins chers mais souvent inefficaces. Nous avons récemment constaté dans huit régions que l’application de telles subventions permet en général une amélioration de la disponibilité des traitements ainsi qu’une réduction de leur prix au détail, mais il est difficile de dire si ces résultats se traduisent par une réelle amélioration de l’utilisation de ces traitements pour les cas suspectés de paludisme.

Méthodes et résultats

Nous avons mené une revue systématique de la documentation pour identifier les rapports expérimentaux ou programmatiques sur les ACT subventionnées permettant ainsi d’évaluer l’impact de ces subventions sur les habitudes des consommateurs. Nous avons analysé les relations entre les prix, les habitudes et les facteurs potentiels de confusion en utilisant des modèles de régression logistique et binomiale ainsi qu’en utilisant une régression linéaire pour souligner l’importance des liens existant entre ces facteurs. Au total, nous avons pu intégrer quarante études à notre analyse, quatorze évaluées par des pairs et vingt-six non évaluées par des pairs. Nous constatons une augmentation sensible des ACT du secteur privé suite à l’application de la subvention. Dans l’ensemble, chaque baisse de prix d’1 US$ est liée à une augmentation de 24% d’achat d’ACT (R_2¼0.302) parmi les cas suspectés de paludisme. De plus ces liens ont pratiquement les mêmes effets sur différents groupes qu’ils soient riches ou pauvres, qu’ils viennent de zones rurales ou urbaines ou qu’ils soient adultes ou enfants.

Conclusions

Les réductions du prix des ACT peuvent entrainer une augmentation de leur utilisation par les personnes suspectées d’avoir contracté le paludisme, et ce même dans les zones les plus reculées où les populations sont le plus exposées au risque de mortalité de cette maladie. Il faut prendre en compte le contexte local, en termes de comportements de recherche de traitements ou de prévalence du paludisme, pour savoir si subventionner est la bonne solution. Les législateurs pourront se servir de cette étude comme base pour leur permettre de prendre des décisions adéquates sur les réductions de prix des ACT afin d’augmenter sensiblement les traitements qui sauvent des vies.

Mots clés : subvention, thérapies combinées à base d'artémisinine, paludisme, revue systématique
