# Supplementary material for: Price subsidies increase the use of private sector ACTs: evidence from a systematic review
Source: Health Policy Plan. 2014 Mar 14;30(3):397–405. doi: 10.1093/heapol/czu013 (PMC4353896; doi:10.1093/heapol/czu013)
Supplement: Translated Abstracts [file supp_czu013_czu013_Spanish.pdf]

Subsidios a los precios aumentan el uso de TCAs del sector privado: la evidencia de una revisión sistemática

Alexandra Morris, Abigail Ward, Bruno Moonen, Oliver Sabot y Justin M. Cohen

Aceptado 4 de febrero de 2014

Antecedentes

Aunque las terapias combinadas basadas en la artemisinina (TCAs) son el tratamiento de primera línea recomendado para el paludismo no complicado en la mayoría de países endémicos, han sido prohibitivamente caras en el sector minorista, donde muchos de los casos sospechosos de malaria compran tratamientos. Los subsidios a las TCAs tratan de estimular la demanda de las drogas por parte de los consumidores por encima de alternativas más baratas, pero a menudo ineficaces, por medio de la reducción de sus precios. La evidencia reciente de ocho regiones que implementan dichos subsidios sugiere que son generalmente exitosos en mejorar la disponibilidad de los medicamentos y disminuir sus precios de venta, pero no queda claro si estos resultados se traducen en un mejor uso por parte de los pacientes con sospecha de malaria.

Métodos y Hallazgos

Se realizó una revisión sistemática de la literatura para identificar los informes de los subsidios experimentales o programáticos de TCA para evaluar el impacto de los subsidios en el uso del consumidor. Las relaciones entre los precios, el uso y los posibles factores de confusión fueron examinadas utilizando medidas logísticas y modelos de regresión de medidas binomiales repetidas y magnitudes aproximadas de asociación se calcularon mediante regresión lineal. En total, 40 estudios, 14 revisados por pares y 26 sin revisión por pares, fueron elegibles para su inclusión en el análisis. Los estudios revisados encontraron un aumento sustancial en el uso de TCA en el sector privado tras la introducción de un subsidio. En general, cada $1 de disminución en el precio estaba relacionado con un aumento de 24 puntos porcentuales en la proporción de casos sospechosos de paludismo que compran los TCA (R^2=0.302). No hubo diferencias significativas evidentes en

esta relación cuando se comparan los grupos más pobres y más ricos, poblaciones rurales y urbanas o niños contra adultos.

Conclusiones
Estos hallazgos sugieren que las reducciones de precios de los TCA pueden aumentar su uso para la sospecha de malaria, incluso dentro de las poblaciones más pobres y más remotas que pueden estar en mayor riesgo de mortalidad por malaria. Si un subsidio es apropiado o no dependerá del contexto local, incluyendo las conductas en la búsqueda de tratamiento y la prevalencia de la malaria. Esta evaluación proporciona una base inicial para que los generadores de políticas tomen decisiones basadas en la evidencia con respecto a la reducción de precios de los TCA para aumentar el uso de medicamentos que potencialmente pueden salvar vidas.

Palabras Claves
Subsidio, terapias combinadas basadas en la artemisinina, paludismo, revisión sistemática

MENSAJES CLAVES

- Los subsidios aumentaron el uso de TCA en el sector privado por niños menores de cinco años en todos los estudios identificados, desde 7 puntos porcentuales en Nigeria a 9 puntos porcentuales en Uganda (ambos a escala nacional).
- En promedio, una reducción de 1 dólar en el precio de los TCAs se relacionó con un aumento de 24 puntos porcentuales en el uso de TCA entre los de cualquier edad, y un incremento de 32 puntos porcentuales entre los niños menores de cinco años.
- Los aumentos observados en el uso de TCA en el sector privado despué́s de los subsidios parecen equitativos; los subsidios aumentaron sustancialmente el uso de TCA incluso entre los individuos estudiados más pobres, y no hubieron diferencias significativas evidentes en el uso de TCA entre los habitantes en zonas rurales frente a zonas urbanas.
